# Yeast NatB Regulates Cell Death of Bax-Expressing Cells

**DOI:** 10.3390/biom15121731

**Published:** 2025-12-12

**Authors:** Joana P. Guedes, Filipa Mendes, Beatriz O. Machado, Stéphen Manon, Manuela Côrte-Real, Susana R. Chaves

**Affiliations:** 1 Centre of Molecular and Environmental Biology (CBMA), Department of Biology, University of Minho, 4710-057 Braga, Portugalfilipamendes06@gmail.com (F.M.); suchaves@bio.uminho.pt (S.R.C.); 2 Institut de Biochimie et Génétique Cellulaires, UMR5095, Centre National de Recherche Scientifique, Université de Bordeaux, 33076 Bordeaux, France; manon@ibgc.cnrs.fr

**Keywords:** Bax, NatB, Bcl-2 family proteins, acetic acid, cell death, *Saccharomyces cerevisiae*

## Abstract

The pro-apoptotic protein Bax is a key apoptosis regulator, as its activity is the main driver of mitochondrial outer membrane permeabilization. Bax is therefore tightly regulated, both by protein–protein interactions and post-translational modifications, such as phosphorylation. Although less studied, N-terminal acetylation has also been implicated in Bax regulation: disruption of the NatB N-terminal acetyl transferase complex in both yeast and MEFs increases Bax mitochondrial localization, although increased translocation is not sufficient to trigger its activation. Using the well-established model of heterologous expression of human Bax in yeast, we further investigated its regulation by N-terminal acetylation. We found that the sensitivity of Bax-expressing cells to acetic acid is greatly enhanced in a strain lacking the yeast NatB catalytic subunit (Nat3p). We propose that the Bax-induced cell death process shifts to a regulated necrosis in this strain due to autophagy inhibition. Furthermore, we show that the protective role of Bcl-xL against acetic acid-induced cell death of Bax-expressing yeast cells requires Nat3p. We speculate that Nat3p modulates the function of pro-death and pro-survival proteins, ultimately affecting both the levels and mode of cell death. These findings may have implications for the development of novel therapeutic strategies targeting human diseases associated with cell death dysfunction.

## 1. Introduction

Apoptosis is a highly regulated cell death mechanism essential to maintain tissue homeostasis. It is mainly controlled by Bcl-2 family proteins, particularly Bax. In healthy cells, Bax predominantly resides in the cytosol but remains in equilibrium with the mitochondrial outer membrane (MOM), where it is loosely bound [1]. In response to apoptotic stimuli, inactive Bax changes from a cytosolic monomeric globular conformation to an extended mitochondrial membrane embedded conformation, followed by protein dimerization and oligomerization. The oligomers assemble into large ring-like structures in the MOM, known as pores, culminating in mitochondrial outer membrane permeabilization (MOMP) (reviewed by [2]). MOMP triggers the release of apoptogenic factors like cytochrome *c* and second mitochondria-derived activator of caspase/direct inhibitor of apoptosis-binding protein (SMAC/DIABLO) from the mitochondria intermembrane space to the cytosol [3]. This event is considered the commitment point in apoptosis and, as such, it is tightly regulated, both by protein–protein interactions, proteolytic cleavage and post-translational modifications [4,5,6,7]. Among the latter, phosphorylation has been the most extensively studied, but N-terminal acetylation (Nt-acetylation) has also been shown to regulate Bax localization and activity [8]. Bax has been identified as an Nt-acetylated protein in human proteomic analyses [9,10] and its N-terminal sequence (MD-) corresponds to a canonical substrate of the NatB N-terminal acetyl transferase complex. NatB is conserved from yeast to mammals and consists of a catalytic subunit (yeast Nat3p/mammalian NAA20) and an auxiliary non-catalytic subunit (yeast Mdm20p/mammalian NAA25). Because the complexity of interacting pathways in mammalian cells complicates the study of Bax regulation by individual modifications, our group recently took advantage of the yeast model system to investigate the molecular mechanisms underlying the regulation of Bax by Nt-acetylation.

The yeast model has been successfully used to study Nt-acetylation, namely to identify new Nt-acetylated proteins, by comparing the level of Nt-acetylation of putative substrates between a wild-type (WT) control strain and a yeast NAT (yNAT)-deleted mutant using proteomics [11,12]. Our group identified Bax as a new yNatB substrate using an In-gel Stable-Isotope Labeling approach [8] to compare the Nt-acetylation levels of human Bax α expressed in WT yeast cells and in a mutant strain lacking the NatB catalytic subunit (*nat3*Δ). Bax was fully Nt-acetylated in WT W303 but not in W303 *nat3*Δ cells, which indicates that ectopically expressed human Bax is Nt-acetylated by Nat3p. We further demonstrated that the Nt-acetylation of Bax is essential to maintain Bax in an inactive conformation in the cytosol of both yeast *nat3*Δ cells and *Naa25*^−/−^ mouse embryonic fibroblasts (MEFs) [8]. Given the conservation of NatB function and the genetic tractability and experimental simplicity of yeast, we used the yeast cell model to further dissect the molecular mechanisms underlying the regulation of Bax by Nt-acetylation [11].

In this study, we show that deletion of *NAT3* increases the sensitivity of Bax-expressing cells to acetic acid, and that this sensitivity is not rescued by expression of the anti-apoptotic protein Bcl-xL. This supports the hypothesis that Nt-acetylation plays an important role in determining both the extent and mode of cell death, highlighting its potential as a therapeutic target in human diseases associated with cell death dysfunctions.

## 2. Materials and Methods

### 2.1. Yeast Strains and Plasmids

The yeast *S. cerevisiae* haploid strain W303-1B (*mat a*, *ade2*, *his3*, *leu2*, *trp1*, *ura3*) was used throughout this study as the WT strain. The W303 *nat3*Δ strain was generated in our previous study [8]. Both strains were transformed with pYES3/CT empty-vector (EV) (Invitrogen, Carlsbad, CA, USA) and pYES3/CT plasmid expressing human WT Bax α, under the control of a *GAL1/10* promoter [13]. For some experiments, the strains were also transformed with pYES2/CT EV (Invitrogen, Carlsbad, CA, USA), pYES2/CT plasmid expressing human Bcl-xL, under the control of a *GAL1/10* promoter [14], or pRS416-GFP-Atg8 [15]. All transformations were performed by the lithium acetate method [16] and the resulting transformants were grown in selective media lacking the appropriate amino acids. Expression levels of Bax α and Bcl-xL in WT and *nat3*Δ cells used throughout this study were verified by Western blot and are shown in Supplementary Materials, Figures S1 and S2.

### 2.2. Growth Conditions

*S. cerevisiae* strains were grown overnight aerobically in synthetic complete (SC) glucose medium [SC; Yeast nitrogen base (0.175%, *w*/*v*) without amino acids and ammonium sulphate, ammonium sulphate (0.5%, *w*/*v*), Drop-out mixture (0.2%, *w*/*v*) and auxotrophic requirements 0.01%] supplemented with glucose (2%, *w*/*v*). The cultures were transferred to SC medium lacking the appropriate amino acids and supplemented with 0.5% (*w*/*v*) of the non-fermentable carbon source lactate/ethanol instead of glucose, to an OD_640nm_ = 0.05 for WT cells and an OD_640nm_ = 0.1 for mutant cells. After approximately 26 h, WT cells were diluted to an OD_640nm_ = 0.25 and mutant cells to an OD_640nm_ = 0.45 in fresh SC lactate/ethanol medium. 5 h later, cultures reached the OD_640nm_ = 0.5 and 2% of galactose (*w*/*v*) was added to trigger the expression of Bax and, in some cases, of Bcl-xL, and the cultures were grown for an additional 14 h. All culture incubations were performed at 30 °C in an orbital shaker at 200 rpm, with a ratio of flask volume/medium of 5:1.

### 2.3. Acetic Acid Treatment

After 14 h of induction of Bax expression, cells were harvested to an OD_640nm_ = 0.5 in SC lactate/ethanol medium and supplemented with 2% galactose (*w*/*v*) at pH 3.0, so that added acetic acid (100 or 160 mM) could enter the cells in its undissociated form (CH_3_COOH), by simple diffusion [17]. Samples were then harvested after 90 and 180 min of incubation. Cells without acetic acid treatment were used as a negative control. All incubations were performed at 30 °C in an orbital shaker at 200 rpm, with a ratio of flask volume/medium of 5:1.

### 2.4. Assessment of Plasma Membrane Integrity

Propidium Iodide (PI, Sigma-Aldrich, St. Louis, MI, USA) was used to assess plasma membrane integrity by flow cytometry and fluorescence microscopy. WT and *nat3*Δ cells expressing Bax α and, in some cases, co-expressing Bcl-xL, were grown and treated as described above. Then, cells were harvested and resuspended in 1× PBS (137 mM NaCl, 10 mM phosphate, 2.7 mM KCl; pH 7.4) containing 2 μM PI and incubated for 10 min at room temperature (RT) in the dark. For visualization, fluorescence was detected by a Leica Microsystems DM-5000B epifluorescence microscope using the 100× oil-immersion objective with the appropriate filter settings for differential phase contrast (DIC) and red fluorescence (N21). For quantification, as *nat3*Δ cells form aggregates, cells were washed twice after PI staining with 1× PBS, resuspended in 1× PBS and then sonicated for 1 min. After confirming the absence of aggregates, the samples were analyzed by flow cytometry. Flow cytometry analysis was performed using a CytoFLEX V0-B4-R2 Flow Cytometer (Beckman Coulter Life Sciences, Indianapolis, IN, USA) equipped with two solid-state lasers (488 nm and 638 nm). The detection of the fluorescence was achieved by collecting red fluorescence through a band-pass filter corresponding to the ECD channel. For each sample, about 10,000 events were collected and analyzed with CytExpert software 2.4 (Beckman Coulter Life Sciences, Indianapolis, IN, USA).

### 2.5. Western Blot Analysis

For preparation of whole cell extracts, 1 mL of cells at an OD_640nm_ = 1 was harvested by centrifugation at 5000× *g* for 3 min and washed once with distilled water. Cells were then resuspended in 500 μL of water containing 50 μL lysis buffer (3.5% (*v*/*v*) β-mercaptoethanol in 2 M NaOH) and incubated for 15 min on ice. Next, 50 μL of 3 M trichloroacetic acid was added and tubes were incubated for 40 min on ice to precipitate the proteins. Extracts were centrifuged at 12,000× *g* for 10 min at 4 °C, washed with 100 μL of acetone and centrifuged again at 12,000× *g* for 5 min at 4 °C. Finally, protein extracts were resuspended in Laemmli buffer (2% SDS, 2% (*v*/*v*) β-mercaptoethanol, 0.1 M Tris pH 8.8, 20% (*v*/*v*) glycerol, 0.02% (*v*/*v*) bromophenol blue). Samples were heated at 70 °C for 15 min. Protein extracts were then separated electrophoretically on a 12.5% sodium dodecyl sulphate-polyacrylamide gel (SDS-PAGE) at 20 mA per gel and transferred to PVDF (GE Healthcare, Chicago，IL, USA) membranes at 60 mA per membrane for 1 h and 30 min. To prevent non-specific binding, the membranes were blocked in 5% (*w*/*v*) non-fat milk in PBS-Tween 0.1% (*v*/*v*) solution (1× PBST) for 1 h at RT with agitation. Afterwards, membranes were incubated overnight with agitation at 4 °C with primary antibodies mouse monoclonal anti-yeast phosphoglycerate kinase (PGK1) antibody (1:5000, Molecular Probes, Eugene, Oregon, EUA), mouse monoclonal anti-yeast porin (POR1) antibody (1:5000, Molecular Probes, Eugene, Oregon, EUA), mouse monoclonal anti-human Bax antibody (1:1000, Santa Cruz Biotechnology, Dallas, TX, USA), anti-GFP antibody (1:3000, Roche, Basel, Switzerland) or rabbit polyclonal anti-human Bcl-xL antibody (1:1000, Cell Signaling, Danvers, MA, USA). After washing with 1× PBST for 15 min, membranes were incubated with the corresponding secondary antibodies against mouse and rabbit IgG peroxidase (1:5000, Sigma-Aldrich) for 1 h at RT with agitation. Immunodetection of bands was revealed by chemiluminescence using the ECL detection system (Millipore-Merck, Burlington, MA, USA) in a ChemiDoc XRS image system with the Quantity One software (BioRad, Hercules, CA, USA). Original Western blot images obtained prior to cropping are shown in Supplementary Materials: Original images.

### 2.6. Statistical Analysis

GraphPad Prism 8.0 (Graph-Pad Software, Inc., La Jolla, CA, USA) was used to perform statistical analysis. The analyses were performed between groups, and the corresponding used test is indicated in the figure legends. The results are exhibited as mean ± S.E.M of at least three independent experiments, and significance was recognized for *p* ≤ 0.05.

## 3. Results

### 3.1. Deletion of NAT3 Increases the Susceptibility of Yeast Cells Expressing Human Bax α to Acetic Acid

**Figure 1:**
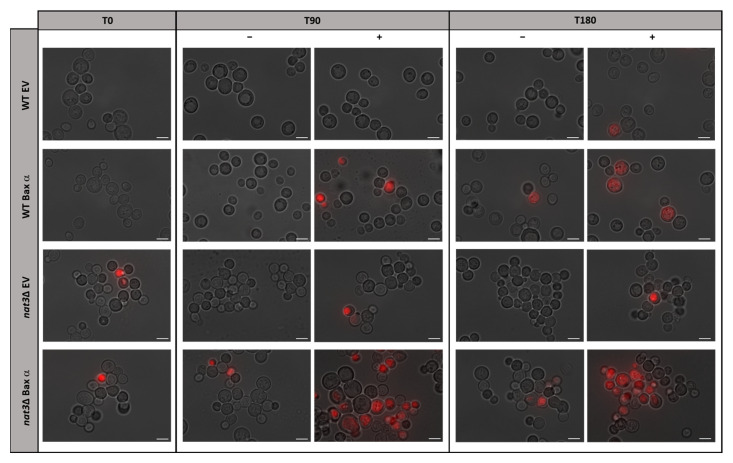
Acetic acid induces loss of plasma membrane integrity in *nat3*Δ yeast cells expressing human Bax α. Fluorescence microscopy images of the indicated yeast strains treated (+) or not (−) with 160 mM acetic acid, pH 3.0, for 0, 90 and 180 min and stained with PI. Strains: W303 cells expressing human Bax α (WT Bax α) or empty vector control (WT EV); *nat3*Δ yeast cells expressing human Bax α (*nat3*Δ Bax α) or empty vector control (*nat3*Δ EV). Expression of Bax was verified by Western blot (Supplementary Materials, Figure S1). The images shown are representative of several microscope fields. Bar: 5 µm.

We first assessed whether Nt-acetylation affects the sensitivity of yeast cells expressing human Bax α to an apoptotic stimulus. For this purpose, acetic acid was used as an exogenous trigger to activate Bax α in both WT and *nat3*Δ cells expressing human Bax α, as we previously showed that yeast cells expressing Bax α displayed increased sensitivity to acetic acid [18]. Because *nat3*Δ cells aggregate [19,20], and therefore viability cannot be assessed by standard colony formation unit (CFU) assays, we evaluated loss of plasma membrane integrity by staining with PI and observing cells by fluorescence microscopy. Exposure of WT and *nat3*Δ cells carrying the empty plasmid to acetic acid did not result in significant staining. However, acetic acid increased PI staining in the strains expressing Bax α, most noticeably in *nat3*Δ cells (Figure 1).

**Figure 2:**
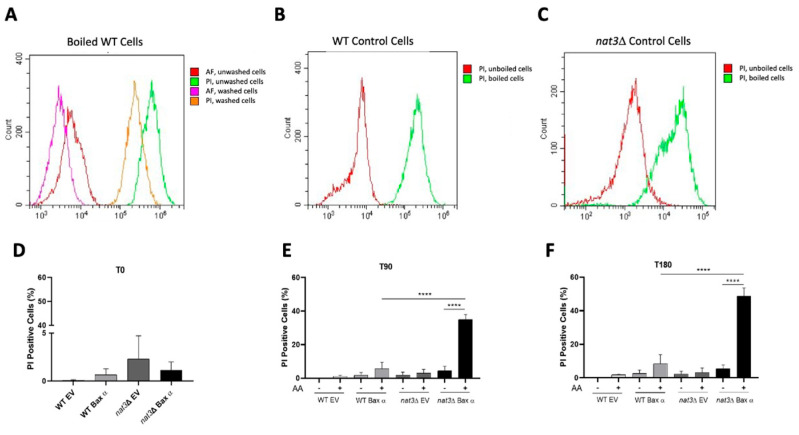
Quantification of acetic acid-induced loss of plasma membrane integrity in WT and *nat3*Δ yeast cells expressing human Bax α, using an optimized PI staining flow cytometry protocol. (**A**) Fluorescence histogram overlays of boiled WT cells (unstained: autofluorescence, AF and PI-stained, PI). Either unwashed or washed after staining. (**B**,**C**) Fluorescence histogram overlays of unboiled and boiled cells after PI staining, washing and sonication of WT (**B**) or *nat3*Δ (**C**) strains. (**D**–**F**) WT or *nat3*Δ strains expressing human Bax α or carrying the empty vector (EV) were treated (+) or not (−) with 160 mM acetic acid, pH 3.0, for 0, 90 and 180 min and stained with PI. The percentage of PI-positive cells was quantified 14 h after Bax expression at T0 (**D**), and after 90 min (T90, **E**) and 180 min (T180, **F**) of treatment. Values were adjusted for autofluorescence (**A**,**F**). Data represent the mean of three independent experiments analyzed by one-way ANOVA (**** *p* < 0.0001; AA—acetic acid).

To quantify these results, we optimized a protocol to monitor loss of plasma membrane integrity of the aggregate-prone *nat3*Δ cells by flow cytometry after PI staining (Figure 2). We first sonicated cells prior to staining with PI to remove aggregates; however, a significant number of PI-negative WT cells became PI-positive (not shown), indicating that sonication itself compromises plasma membrane integrity. This confirms that sonication is unsuitable for this method or for separating aggregates prior to CFU assays. We therefore developed an alternative strategy in which cells were first stained with PI, unbound probe washed away, and only then were cells sonicated. To evaluate this approach, we boiled WT cells to compromise plasma membrane integrity, stained them with PI, and compared washed *versus* unwashed samples by flow cytometry. We found that washing decreased fluorescence intensity but did not alter the percentage of PI-stained cells (Figure 2A). Second, we assessed if sonication interferes with PI staining by comparing the percentage of PI-positive boiled WT cells that were washed and subsequently sonicated (Figure 2B) with those that were washed only (Figure 2A). Sonication did not alter the percentage of PI-positive WT cells, validating the protocol alterations. With these conditions established, we applied the optimized protocol to *nat3*Δ cells (Figure 2C). Boiled (PI-positive) and unboiled (PI-negative) *nat3*Δ cells remained clearly distinguishable, confirming the method reliably discriminates between cells that lost plasma membrane integrity from those that did not.

Using the optimized method, we observed only a small percentage of WT and *nat3*Δ cells stained with PI 14 h after induction of Bax expression, indicative of minimal basal cell death (Figure 2D). 90 min of exposure to acetic acid significantly increased the percentage of PI-positive *nat3*Δ cells expressing Bax α, which slightly increased after 180 min of acetic acid treatment; in contrast, there was only a minor increase in the percentage of PI-positive WT cells expressing Bax (Figure 2E,F). As expected, no toxicity was observed in either WT or *nat3*Δ cells transformed with the EV, either untreated or treated with acetic acid. Altogether, fluorescence microscopy and flow cytometry results demonstrate that the absence of Nat3p-mediated Nt-acetylation renders yeast cells more sensitive to acetic acid, as assessed by loss of plasma membrane integrity.

### 3.2. The Increased Susceptibility of nat3Δ Yeast Cells Expressing Human Bax α to Acetic Acid Is Associated with Autophagy Inhibition

**Figure 3:**
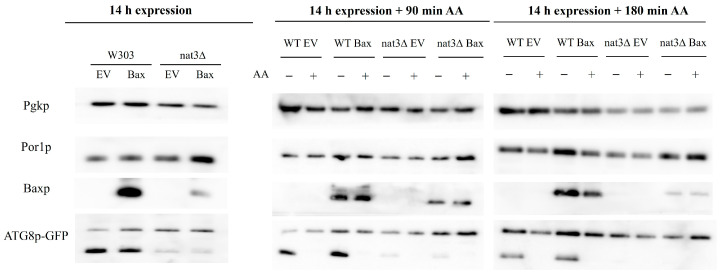
Absence of Nat3p decreases autophagy. WT and *nat3*Δ cells harboring pRS416 GFP-ATG8 and transformed with the EV or expressing Bax α were grown in galactose for 14 h and then treated (+) or not (−) with 160 mM acetic acid, pH 3.0. Samples were collected before treatment (time 0) and after 90 and 180 min. Autophagy was monitored by western-blot analysis of GFP-Atg8 cleavage. Diminished cleavage of Atg8-GFP in *nat3*Δ cells at this time point was observed consistently (*n* = 3) and also observed in starvation media (not shown). Pgk1p was used as the loading control. The original Western blot images are shown in Supplementary Materials: Original images.

It has previously been shown that inhibition of autophagy accelerates Bax-induced loss of plating efficiency in yeast cells expressing a constitutively active form of Bax (c-myc), associated with a transition to a necrotic type of cell death [21]. We therefore sought to investigate whether the high susceptibility to acetic acid observed in *nat3*Δ cells expressing Bax α could result from impaired autophagy, which has been reported in another *nat3*Δ strain [22]. To this end, autophagy induction was assessed in WT and *nat3*Δ cells expressing Bax α by monitoring cleavage of co-expressed Atg8-GFP. We found that autophagy was clearly induced 14 h after galactose addition in WT but not *nat3*Δ cells (Figure 3; original WB images are in Supplementary Materials: Original images). In accordance with our previous study [23], exposure to acetic acid inhibited autophagy in WT cells (Figure 3). In contrast, mitophagy was not induced under our experimental conditions (Supplementary Materials, Figure S3).

### 3.3. Bcl-xL Does Not Rescue nat3Δ Yeast Cells Expressing Human Bax α from Acetic Acid-Induced Cell Death

**Figure 4:**
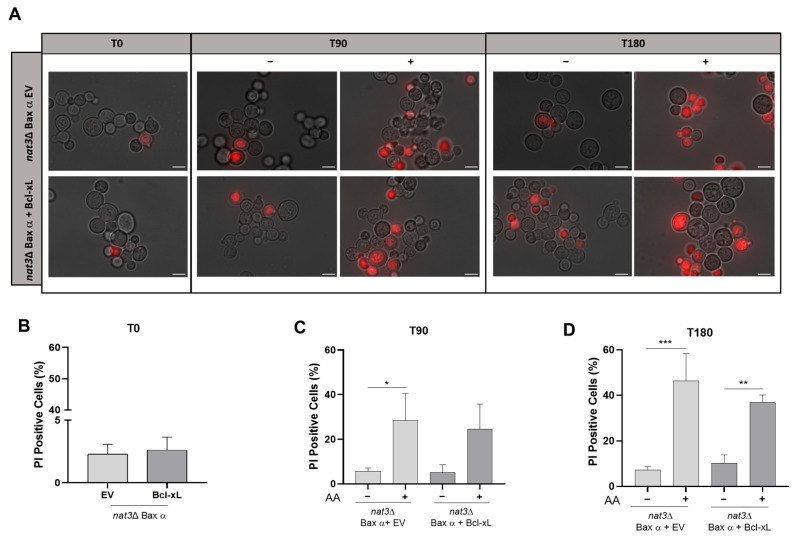
Bcl-xL does not rescue *nat3*Δ yeast cells expressing human Bax α from acetic acid-induced loss of plasma membrane integrity. Plasma membrane integrity was assessed by PI staining of *nat3*Δ yeast cells co-expressing human Bax α and Bcl-xL or the respective empty vector (EV). Expression of the respective proteins was verified by Western blot (Supplementary Materials, Figure S2). (**A**) Fluorescence microscopy images of the indicated yeast strains treated (+) or not (−) with 160 mM acetic acid, pH 3.0, for 0, 90 and 180 min and stained with PI. Images shown are representative of several microscope fields. Bar: 5 µm. (**B**–**D**) Representative plots of PI-positive stained cells (**B**) 14 h after Bax and Bcl-xL expression without treatment, (**C**) after 90 min and (**D**) after 180 min of treatment without (−) or with (+) 160 mM acetic acid, pH 3.0. The percentage of PI-positive cells was adjusted to autofluorescence. Values represent the mean of three independent experiments analyzed by one-way ANOVA (* *p* < 0.05, ** *p* < 0.01 and *** *p* < 0.001. AA—acetic acid).

As Bcl-xL protects WT yeast cells expressing Bax α from acetic acid-induced cell death [18], we sought to address whether Bcl-xL could also revert the susceptibility of *nat3*Δ cells to acetic acid. To this end, we monitored the plasma membrane integrity of *nat3*Δ cells heterologously co-expressing Bax α and Bcl-xL or the respective EV at 90 and 180 min after exposure to acetic acid. Fluorescence microscopy showed that *nat3*Δ cells expressing Bax α exhibited similar levels of PI-positive stained cells after acetic acid treatment regardless of Bcl-xL co-expression (Figure 4A). Flow cytometry analysis to quantify the phenotype confirmed these results: there was a residual percentage of PI-positive cells 14 h after Bax α expression, either alone or with co-expression of Bcl-xL (Figure 4B), and this percentage increased to a similar extent after 90 and 180 min of acetic acid treatment (Figure 4C,D).

Altogether, fluorescence microscopy and flow cytometry analyses suggest that Bcl-xL does not protect *nat3*Δ cells expressing Bax α from acetic acid-induced cell death, as there was no significant difference in the percentage of cells with compromised plasma membrane integrity compared to cells not expressing Bcl-xL. This suggests that the absence of Nt-acetylation may interfere with the interaction between Bax and Bcl-xL, compromising the anti-apoptotic role of Bcl-xL, or that Bax-induced cell death in this strain is no longer apoptotic in nature. We recently described that acetic acid-induced PI staining increases after washing cells and re-suspending them in stimulus-free medium, when a regulated cell death process associated with early preservation of plasma membrane integrity is induced [24]. To explore whether this was the case here, we monitored plasma membrane integrity of *nat3*Δ cells expressing Bax α 180 min after exposure to 100 mM or 160 mM of acetic acid, and again 2 and 4 h after washing cells and transferring to fresh medium. The percentage of PI-positive cells remained similar during the recovery period, for either acetic acid concentration. These results support the hypothesis of a transition to a cell death process associated with an early loss of plasma membrane integrity, typical of necrosis, in this strain background (Supplementary Materials, Figure S4).

## 4. Discussion

We have previously shown that Nt-acetylation influences Bax α localization, observing increased mitochondrial addressing in both in *nat3*Δ yeast and *Naa25*^−/−^ MEFs [8]. However, this increased mitochondrial localization in the absence of NatB-mediated acetylation was not sufficient to induce cell death. We later reported that yeast cells expressing inactive human Bax α die upon exposure to sub-lethal doses of acetic acid, and that this process is rescued by Bcl-xL expression, indicating that activation of untagged, non-mutated Bax α can be recapitulated in yeast using an exogenous stimulus [18]. Studies reporting expression of inactive human Bax α, unable to kill yeast cells, are scarce, as most focus on Bax activity and therefore rely on conditions where the protein is active. In many cases, this is achieved by expressing tagged forms of Bax, which alter conformations of the N- and C-terminal regions that are crucial to its activation. In particular, Bax activation is caused by destabilization of the C-terminal helix α9, favoring its extrusion from the hydrophobic groove (see also [25,26]). Because our objective was to dissect the specific contribution of NatB-mediated Nt-acetylation to Bax regulation, we expressed unmodified, inactive Bax α in a strain lacking the yeast NatB catalytic subunit (Nat3p), enabling its controlled activation by acetic acid.

Since the *S. cerevisiae* genome lacks orthologs of the human Bcl-2 family, it was possible to address the effect of Nt-acetylation on Bax regulation, without interference from the mammalian apoptotic network. Using this model, we showed that absence of Nat3p-mediated Nt-acetylation greatly increases the sensitivity of yeast cells expressing Bax α to death induced by acetic acid, as assessed by increased PI staining indicative of loss of plasma membrane integrity. Furthermore, Bcl-xL could not protect Bax α-expressing *nat3*Δ cells from acetic acid-induced loss of plasma membrane integrity, in contrast to its protective effect when Nt-acetylation is intact [18]. It is known that Bcl-xL can counteract Bax α activation by forming inhibitory complexes in mitochondria or promoting the retrotranslocation of Bax α to the cytosol [27]. Further studies are required to elucidate if loss of Bax α Nt-acetylation compromises one or both of these protective mechanisms.

The increased sensitivity of Bax α-expressing *nat3*Δ cells to death induced by acetic acid is in agreement with findings in mammalian systems. For instance, inhibition of the catalytic subunit of NatB (*NAA20*) in HeLa cells sensitizes cells to apoptosis induced by the proteasome inhibitor MG132 [28]. Also, our previous study showed that MEFS lacking the regulatory subunit of NatB (*Naa25*^−/−^ MEFs) are more sensitive to MG132 [8]. Similarly, a recent study found that NatB plays an important role in stress prevention [29]. It has previously been shown that activation of Bax, either through mutation, C-terminal tag or acetic acid treatment, leads to loss of plating efficiency with apoptotic-like and/or autophagic features, but without loss of plasma membrane integrity [18,21]. After plating on media repressing Bax expression, cells continue the cell death process, then failing to give rise to colonies on solid media. A higher percentage of cell death by colony forming units than the percentage of PI-positive cells at the time of stimulus removal reflects a yeast regulated cell death (yRCD) process [30], with cells eventually losing plasma membrane integrity, designated by some authors as secondary necrosis [21,31]. We sought to investigate whether the increased PI staining observed in *nat3*Δ cells expressing Bax α exposed to acetic acid reflected a faster RCD, followed by secondary necrosis, or instead a shift toward an early loss of plasma membrane integrity typical of a yeast necrosis regulated cell death (yN-RCD) or accidental cell death (ACD). However, it was not possible to directly compare loss of viability of *nat3*Δ cells assessed by colony-formation unit (CFU) counts with the percentage of PI-positive cells. Nonetheless, several observations support the hypothesis of yN-RCD. On one hand, we did not observe an increase in the percentage of PI-positive cells after washing cells and further growth in media without acetic acid, suggesting that loss of plasma membrane integrity occurred ab initio rather than progressively during a post-stimulus phase. Indeed, we previously showed that an increase in the PI-positive population during recovery is associated with an yA-RCD process [24]. Moreover, the fact that the increase in the loss of plasma membrane integrity is gene-dependent, in this case Nat3p-dependent, argues against the possibility of ACD, which is not genetically controlled.

While MOMP is the crucial event in apoptosis that leads to the release of apoptogenic factors, in necrosis it is the permeabilization of the inner mitochondrial membrane through the opening of the mitochondrial permeability transition pore (MPTP) that causes a reduction in ATP and impairment of caspase activation, despite the accumulation of apoptogenic substrates like cytochrome *c* in the cytosol. The classical model of the mitochondrial permeability transition pore (MPTP) proposed that it spanned both the outer and inner mitochondrial membranes, involving the adenine nucleotide translocator (ANT) and the voltage-dependent anion channel (VDAC), regulated by cyclophilin D (CypD) within the mitochondrial matrix [32,33]. However, genetic studies showed that neither ANT [34] nor VDAC [35] are essential for pore formation, mitochondrial swelling, or necrotic cell death. In contrast, deletion of CypD confirmed its role in regulating MPTP opening [36,37]. Later evidence pointed to the mitochondrial F_1_F_0_ ATP synthase as the core component of the MPTP within the inner membrane [38,39,40]. The pro-death members of the Bcl-2 protein family Bax and Bak were early recognized to be the main activators of MOMP but were unexpectedly later shown to also play a role in non-apoptotic death [41]. Cells and isolated mitochondria lacking Bax/Bak were resistant to MPTP opening and necrosis, but this phenotype was reverted by reconstitution with wild-type Bax, or an oligomerization-deficient mutant that cannot support MOMP and apoptosis [42]. Thus, Bax and Bak were found to be necessary for outer mitochondrial membrane rupture during necrosis, independent of their usual activation and oligomerization involved in MOMP and apoptosis. Altogether, these data indicate that Bax-regulated apoptosis and necrosis occur through distinct mechanisms. As discussed above, sub-lethal concentrations of acetic acid induce human Bax α mitochondrial translocation and cytochrome *c* release in yeast cells, yet the standard Bax active conformation was not detected [18]. One hypothesis proposed by the authors was that increased accumulation of Bax, either inactive or in a not already characterized active conformation, leads to MOMP and subsequent cytochrome *c* release. However, cytochrome *c* can also be released due to changes in the outer mitochondrial membrane’s physical properties caused by inactive Bax, increasing membrane permeability and rendering mitochondria more prone to rupture after MPTP opening and necrosis [42]. Considering these observations, it would be interesting to address whether the increase in the loss of plasma membrane integrity of *nat3*Δ cells expressing Bax in response to acetic acid primarily reflects rupture of the inner mitochondrial membrane mediated by MPTP opening, rather than classical MOMP associated with preservation of inner mitochondria membrane integrity.

A similar phenotype of increased loss of plasma membrane integrity was observed in cells lacking Uth1p expressing c-myc-tagged Bax, a constitutively active form of Bax that induces loss of plating efficiency (assessed by CFU counts) in yeast cells not normally associated with the loss of plasma membrane integrity [21]. In the *uth1*Δ strain, even though loss of CFUs decreased, loss of plasma membrane integrity increased. Authors therefore concluded that Uth1p was required for a regulated cell death process associated with preservation of plasma membrane integrity. Uth1p has been implicated in autophagic degradation of mitochondria, although it is not required for autophagy induction *per se* [43,44,45]. In our study, we did not observe mitophagy induction along the experimental conditions tested, suggesting the involvement of a different process. Kiššová and colleagues also reported that inhibition of autophagy by deletion of certain *ATG* genes increased the loss of CFUs as a result of Bax-c-myc expression, but without increasing plasma membrane permeability [21]. Therefore, there seems to be evidence that alterations in different steps of autophagy impact the cell death mechanism triggered by active Bax. It has been reported that *nat3*Δ cells are deficient in autophagy, since GFP-Atg8 cleavage in response to starvation is abolished in this strain [22]. We therefore monitored GFP-Atg8 cleavage by Western blot in WT and *nat3*Δ cells expressing Bax α before and after treatment with acetic acid. As expected, we found that there was no GFP-Atg8 cleavage in the presence of acetic acid, in either WT or *nat3*Δ cells. However, prior to acetic acid exposure, WT cells displayed increased autophagy after Bax α expression, whereas *nat3*Δ cells did not. These results suggest that inhibition of autophagy prior to Bax activation precludes cells from executing an RCD process associated with preservation of plasma membrane integrity, although other mechanisms cannot be excluded. Indeed, it was shown that the yeast vacuolar protease Pep4p, ortholog of the human Cathepsin D, has a cytoprotective role both against apoptosis and necrosis that depends on proteolytic activity [46,47] or on the proteolytically inactive propeptide, respectively [46]. The anti-necrotic role of the Pep4p propeptide, which induced histone hypoacetylation, relied on polyamine biosynthesis. This effect was mediated through elevated intracellular levels of putrescine, spermidine, and the precursor S-adenosyl-methionine. In our previous studies, we found that non-lethal doses of acetic acid in cells expressing a cytosolic inactive form of human Bax triggered a Pep4p-inhibitable cell death, since Bax-induced cell death was enhanced in the absence of Pep4p [48]. In addition, we also showed that mouse embryonic fibroblast cells lacking NatB display reduced Nt-acetylation and protein accumulation of some of its substrates, including spermidine synthase [49]. Thus, in the future, it would also be interesting to address the hypothesis that enhanced cell death of *nat3*Δ cells expressing Bax by acetic acid, monitored by a large increase in plasma membrane integrity loss, may be due to a decrease in intracellular spermidine and ensuing deficit of the Pep4p propeptide protective role against necrosis.

## 5. Conclusions

The results gathered in this study demonstrate that the sensitivity of Bax-expressing yeast cells to acetic acid, as well as its suppression by Bcl-xL, depends on NatB. This indicates that Nt-acetylation plays a crucial role in determining both the mode of cell death triggered by Bax and also its regulation. Altogether, our data uncover a previously unrecognized layer of human Bax α regulation that may provide novel opportunities for its therapeutic modulation in the context of human pathologies associated with cell death dysfunctions.

## Data Availability

The original contributions presented in this study are included in the article/Supplementary Materials. Further inquiries can be directed to the corresponding author.

## Supplementary Materials

The following supporting information can be downloaded at: https://www.mdpi.com/article/10.3390/biom15121731/s1. Figure S1: Western blot analysis of WT and *nat3*Δ cells transformed with the EV or a plasmid expressing Bax α 14 h after induction of Bax expression with galactose. Figure: S2. Western blot analysis of *nat3*Δ cells co-expressing Bax α and Bcl-xL or the respective EV. Figure S3. Assessment of mitophagy. Figure S4. Assessment of plasma membrane integrity of Bax-expressing *nat3*Δ cells exposed to acetic acid, before and after stimulus removal. Original images for the Western blots.
