# Supplementary material for: Yeast NatB Regulates Cell Death of Bax-Expressing Cells
Source: Biomolecules. 2025 Dec 12;15(12):1731. doi: 10.3390/biom15121731 (PMC12730891; doi:10.3390/biom15121731)
Supplement: Supplementary file 1 [file biomolecules-15-01731-s001.zip › Supplementary Figures.pdf]

SUPLEMMENTARY FIGURES

Yeast NatB Regulates Cell Death of Bax-expressing Cells

Joana P. Guedes[1], Filipa Mendes[1], Beatriz Machado[1], Stéphen Manon[2], Manuela Côrte-Real[1]*, Susana R. Chaves[1]

1. Centre of Molecular and Environmental Biology (CBMA), Department of Biology, University of Minho, Braga, Portugal

2. UMR 5095 CNRS/Université de Bordeaux, Bordeaux, France

* corresponding author

Manuela Côrte-Real mcortereal@bio.uminho.pt

Department of Biology, University of Minho

Campus de Gualtar, 4710 – 057 Braga, Portugal

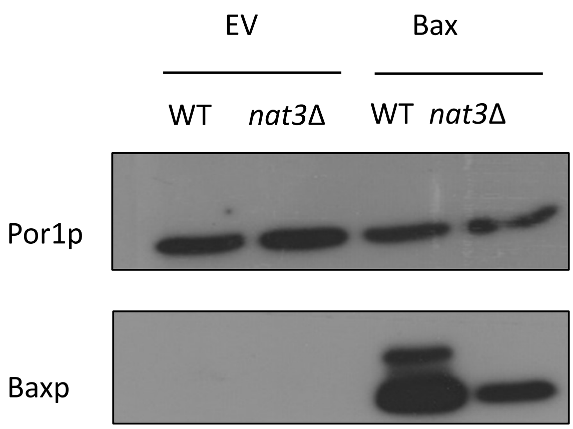

Figure S1. Western blot analysis of WT and nat3Δ cells transformed with the EV or a plasmid expressing Bax α 14h after induction of Bax expression with galactose. Por1p was used as the loading control.

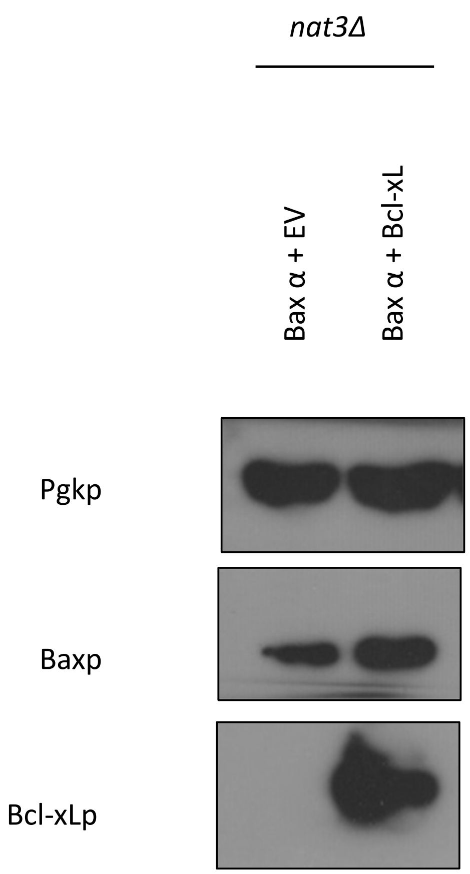

Figure S2. Western blot analysis of *nat3*Δ cells co-expressing Bax α and Bcl-xL or the respective EV. Pgk1p was used as the loading control.

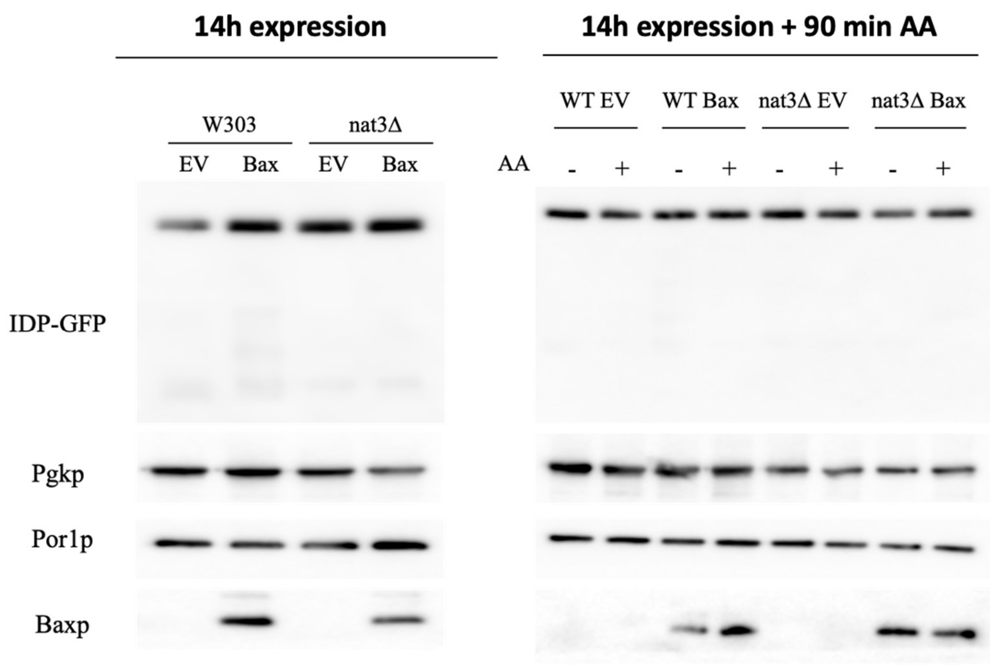

Figure S3. Assessment of mitophagy. WT and *nat3Δ* cells harboring pRS416 IDP-GFP and transformed with the EV or Bax α 14h after induction of Bax expression with galactose were treated or not with acetic acid. Samples were collected before (time 0) and after 90 min of treatment without (-) or with (+) 160 mM acetic acid, pH 3.0. Mitophagy was monitored by western-blot analysis of IDP-GFP cleavage. No cleavage was observed under these experimental conditions; data represent one experiment. Pgk1p was used as the loading control.

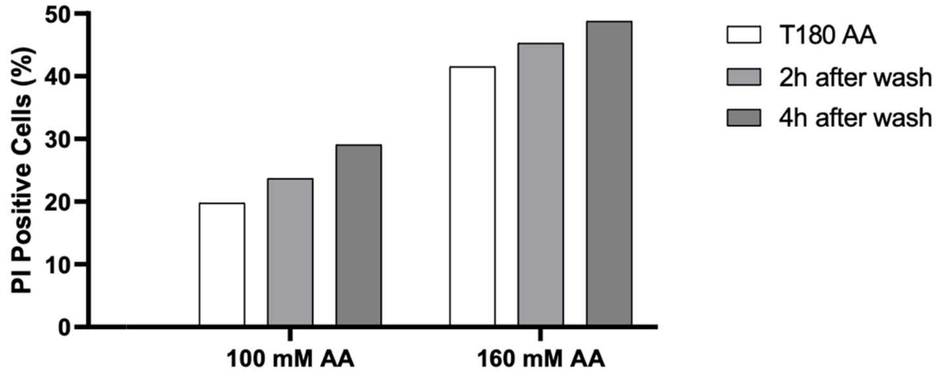

Figure S4. Assessment of plasma membrane integrity of Bax-expressing *nat3Δ* cells exposed to acetic acid, before and after stimulus removal. *nat3Δ* cells expressing Bax for 14h were treated with 100 or 160 mM acetic acid, pH 3.0, for 180 min. After, cells were washed and resuspended in fresh medium without acetic acid for 2 and 4h. Samples were stained with 4 mg/mL of PI for 10 min at RT in the dark. The percentage of PI-positive cells was quantified 14 h after Bax expression followed by 180 min of treatment with the indicated acetic acid

concentrations ("T180AA"), and 2h or 4h after transfer to fresh medium ("2h after wash" and "4h after wash", respectively). The percentage of PI positive cells was adjusted to that at time 0 of acetic acid treatment (i.e., 14 h after Bax expression). Data represent 2 experiments, one with each concentration.
