# Supplementary material for: Yeast NatB Regulates Cell Death of Bax-Expressing Cells
Source: Biomolecules. 2025 Dec 12;15(12):1731. doi: 10.3390/biom15121731 (PMC12730891; doi:10.3390/biom15121731)
Supplement: Supplementary file 1 [file biomolecules-15-01731-s001.zip › Supplementary-Original Western Blot images.pdf]

SUPLEMMENTARY MATERIALS: ORIGINAL IMAGES

Yeast NatB Regulates Cell Death of Bax-expressing Cells

Joana P. Guedes[1], Filipa Mendes[1], Beatriz Machado[1], Stéphen Manon[2], Manuela Côrte-Real[1]*, Susana R. Chaves[1]

1. Centre of Molecular and Environmental Biology (CBMA), Department of Biology, University of Minho, Braga, Portugal

2. UMR 5095 CNRS/Université de Bordeaux, Bordeaux, France

* corresponding author

Manuela Côrte-Real mcortereal@bio.uminho.pt

Department of Biology, University of Minho

Campus de Gualtar, 4710 – 057 Braga, Portugal

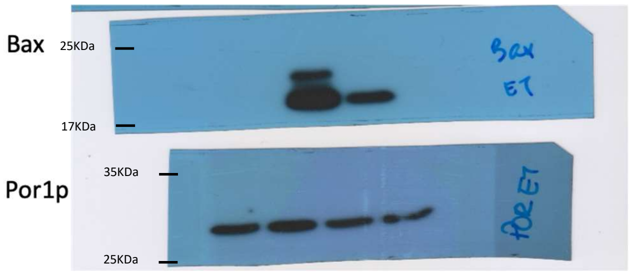

Original gel image shown in Suppl. Figure S1. Western blot analysis of WT and *nat3*Δ cells transformed with the EV or a plasmid expressing Bax α 14h after induction of Bax expression with galactose. Por1p was used as the loading control. Approximate Molecular weight Markers are shown.

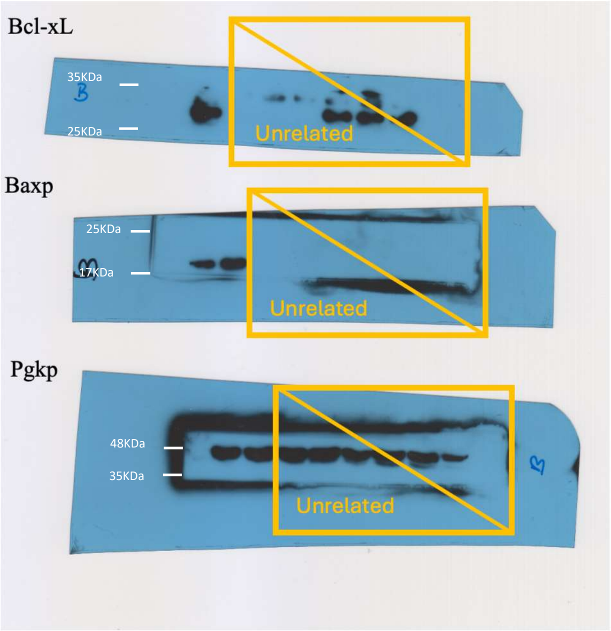

Original gel image shown in Suppl. Figure S2. Western blot analysis of *nat3*Δ cells co-expressing Bax α and Bcl-xL or the respective EV. Pgk1p was used as the loading control. Approximate Molecular weight Markers are shown.

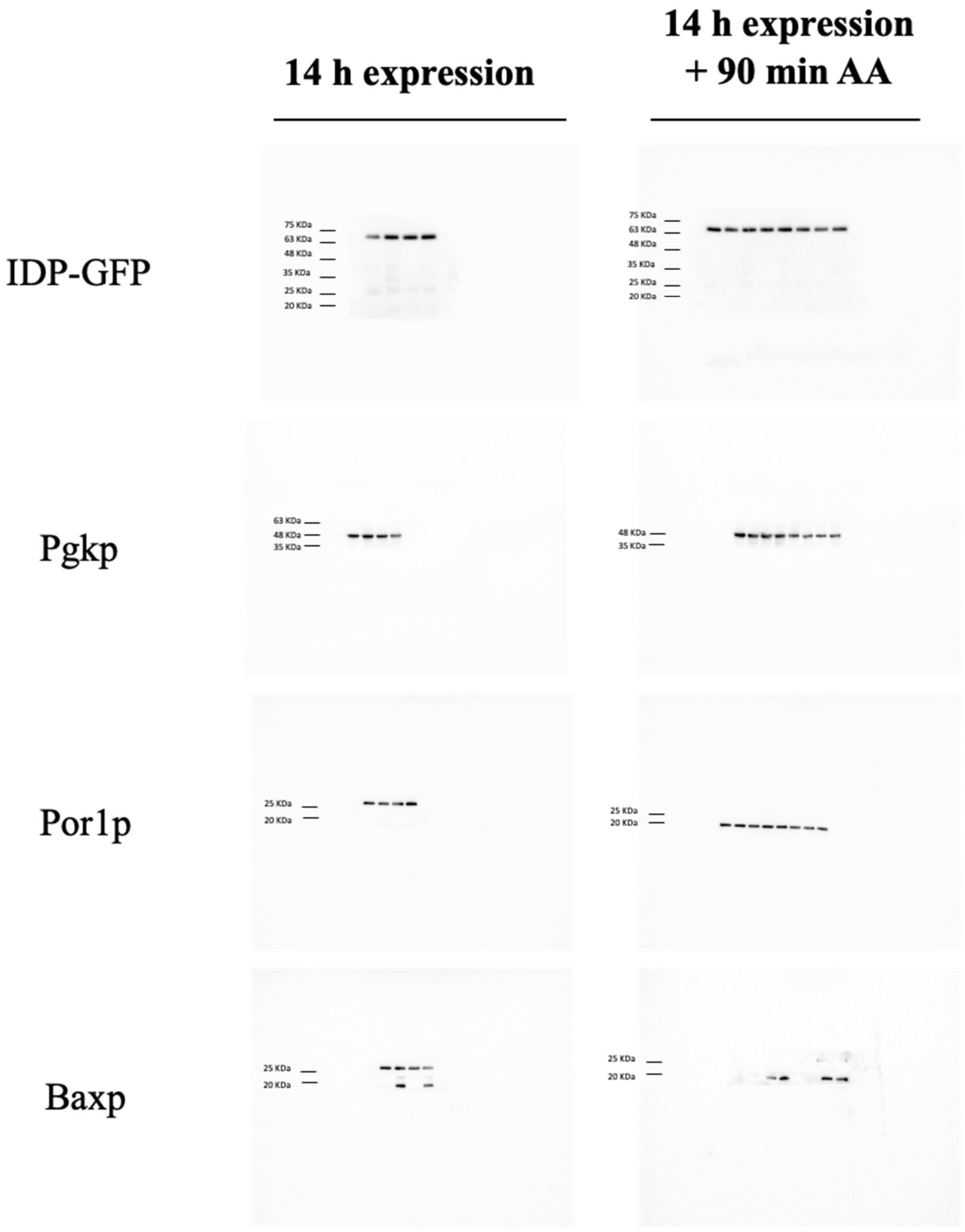

Original gel image shown in Suppl. Figure S3. WT and *nat3*Δ cells harboring pRS416 IDP-GFP and transformed with the EV or Bax α 14h after induction of Bax expression with galactose were treated or not with acetic acid. Samples were collected before (time 0) and after 90 min of treatment without (-) or with (+) 160 mM acetic acid, pH 3.0. Mitophagy was monitored by western-blot analysis of IDP-GFP cleavage. Pgk1p was used as the loading control. Approximate Molecular weight Markers are shown.

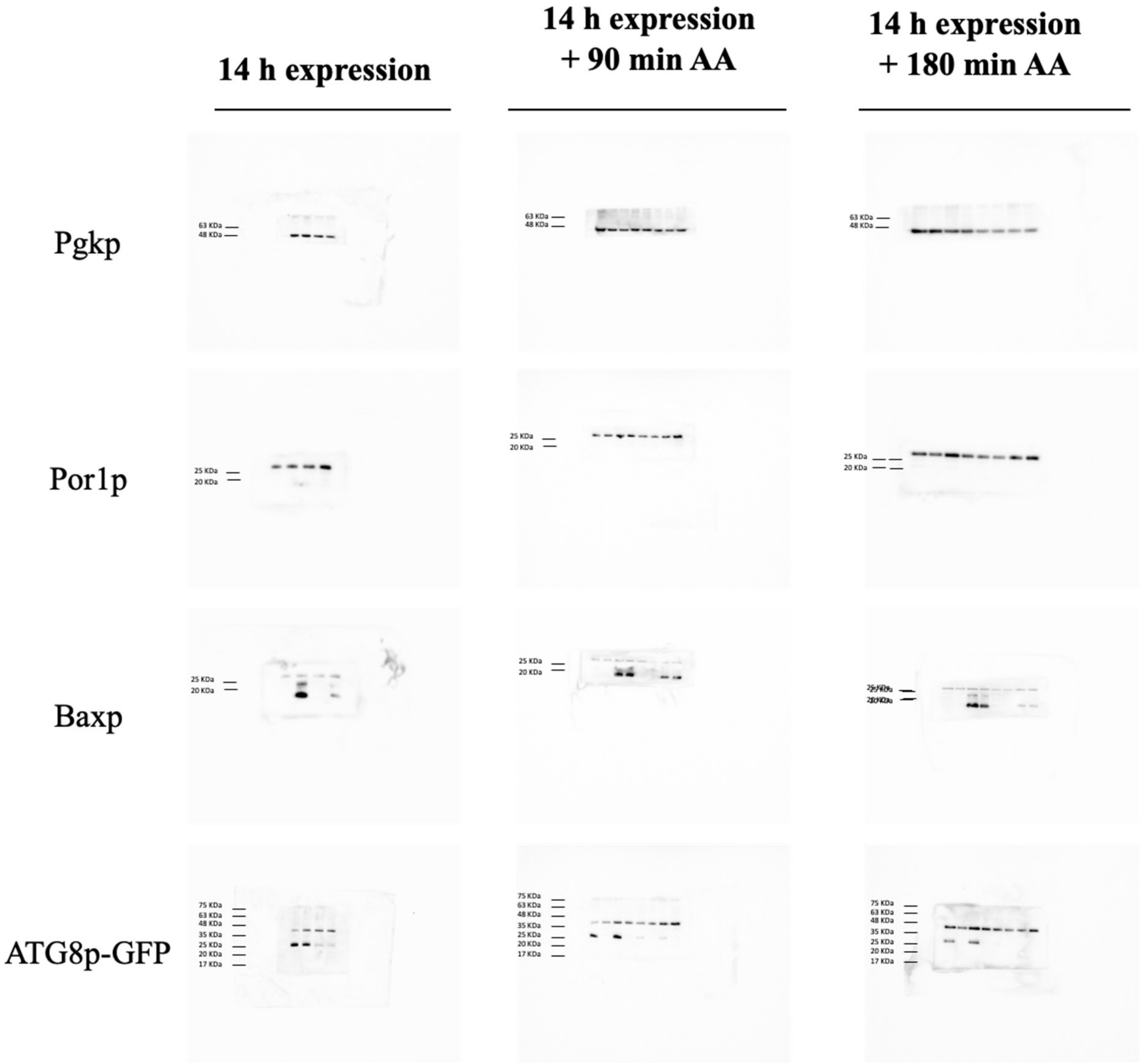

Original blot images of blots shown in Figure 3. Absence of Nat3p decreases autophagy. WT and nat3Δ cells harboring pRS416 GFP-ATG8 and transformed with the EV or expressing Bax α were grown in galactose for 14h and then treated (+) or not (-) with 160 mM acetic acid, pH 3.0. Samples were collected before treatment (time 0) and after 90 and 180 min. Autophagy was monitored by western-blot analysis of GFP-Atg8 cleavage. Pgk1p was used as the loading control. Approximate Molecular weight Markers are shown.
